# The COVID-19 pandemic and mental health in pregnant women: A review article

**DOI:** 10.3389/fpsyt.2022.949239

**Published:** 2022-09-20

**Authors:** Niloufar Arzamani, Shiva Soraya, Fatemeh Hadi, Sara Nooraeen, Mahdieh Saeidi

**Affiliations:** ^1^Mental Health Research Center, School of Behavioral Sciences and Mental Health, Iran University of Medical Sciences, Tehran, Iran; ^2^Research Center of Addiction and Risky Behaviors, Department of Psychiatry, School of Medicine, Iran University of Medical Sciences, Tehran, Iran

**Keywords:** pregnancy, COVID-19, mental health, anxiety, depression

## Abstract

A rapid spreading of the COVID-19 virus in recent years had a great impact on every single aspect of live and the world faced with unexpected and unpredictable crisis in both physical and mental condition. As with any crisis, vulnerable individuals like pregnant women were the concern of societies. Several physiological and psychological changes occur during pregnancy which put individuals in a risk of mental health problems. During the outbreak of the COVID-19, pregnant women have experienced more psychological stresses, fear, anxiety, and depression. The prenatal mental distresses and psychiatric disorders may cause poor compliance, reduce help-seeking behaviors, and neglect to take the follow up screening visits and risk of harm for mother and others. Addressing the mental health in pregnant women is crucial to prevent the consequences. The purpose of this narrative review was to investigate the available literature on the impact of the COVID-19 pandemic on mental health in pregnant women and provide some recommendations to improve mental health in them. It also shed some light on providing mental health services for women during pregnancy and can be used by health professionals and policymakers.

## Introduction

In late December 2019, a novel infection has been reported in Wuhan, China. The COVID-19 spreads rapidly around the world. On January 12, 2020, the World Health Organization (WHO) announced the coronavirus disease 2019 (COVID-19), as a global pandemic with ultra-rapid mortality and morbidity rate (1). High transmission rate among people and the absence of proper knowledge about the nature of the pathogenesis, lack of concise and comprehensive treatment and approved vaccines make governments impose mandatory public health policies, mobility restrictions, and stay-at-home orders to reduce the transmission. Prolonged social and physical distancing and uncertainty about the future and multitude changes that the COVID-19 brings, leads to distresses and affect mental health and quality of life (2, 3). Mental health crisis during the COVID-19 pandemic brings a multitude of psychological distresses and emotional burdens, and people faced unexpected fear and anxiety about their future and family members' physical condition. Exacerbation of depressive symptoms, obsessional thoughts, compulsive behaviors and other pre-existing psychiatric disorders, a sharp rise in domestic violence has been reported during the pandemic. Meanwhile, pregnant women as vulnerable individuals and their mental health is a public health priority and needs special consideration during the crisis (4–8).

Pregnancy is a unique maternal experience with both blesses and distress. The rapid hormonal changes in women's bodies put them in an emotionally unstable situation and they faced more fear, anxiety, and mood changes (9–14). There is an increased risk for anxiety and depression during pregnancy, and they are more susceptible to depressive disorder with peripartum onset (15, 16). Although the risk of COVID-19 infection is not more in pregnant women, the fear of getting infected among them is high (17).

Past medical and psychiatric history, genetic predisposition, lack of proper family support, prenatal complications, and stressful life events may affect the prenatal mental health. It has been widely investigated that the endocrine system has a key role in different changes during pregnancy and influence on behavioral and affective status of pregnant women (18).

Depressive symptoms in pregnancy have been linked to the dysregulation of cortisol production. High levels of maternal cortisol affects fetus' health (19). The prevalence of generalized anxiety disorder (GAD) in pregnant women has been reported 3–4 times greater than in the general population during the COVID-19 pandemic. Studies had shown that the rate of anxiety and depression among pregnant women during the COVID-19 pandemic had raised (20). A review article reported that the anxiety and depressive symptoms were highly prevalent, effecting 58–72% of pregnant women during the COVID-19 pandemic (20).

Pregnancy has a significant physiological, psychological, and biochemical effects on women's life. Addressing the importance of mental health during pregnancy is crucial for the mother's wellbeing, and reaching the neurodevelopmental milestone of the infant (21).

In this review we conducted systematic searches of the literature in order to address the pregnant women's mental health during the COVID-19 pandemic and deliver some recommendations to improve their mental health.

## Search strategy and selection criteria

This narrative review study was conducted using related articles available in valid English scientific databases such as PubMed, Scopus, Web of Science, and Google scholar, which were published from 2020 to 2022. The keywords were (((COVID^*^[Title]) OR (Coronavirus[Title])) AND ((pregnant^*^[Title]) OR (perinatal[Title]) OR (maternal[Title]) OR (pre labor[Title])) AND ((mental[Title/Abstract]) OR (Anxiety[Title/Abstract]) OR (worry^*^[Title/Abstract]) OR (depression ^*^[Title/Abstract]) OR (fear[Title/Abstract]))).

**Figure 1:**
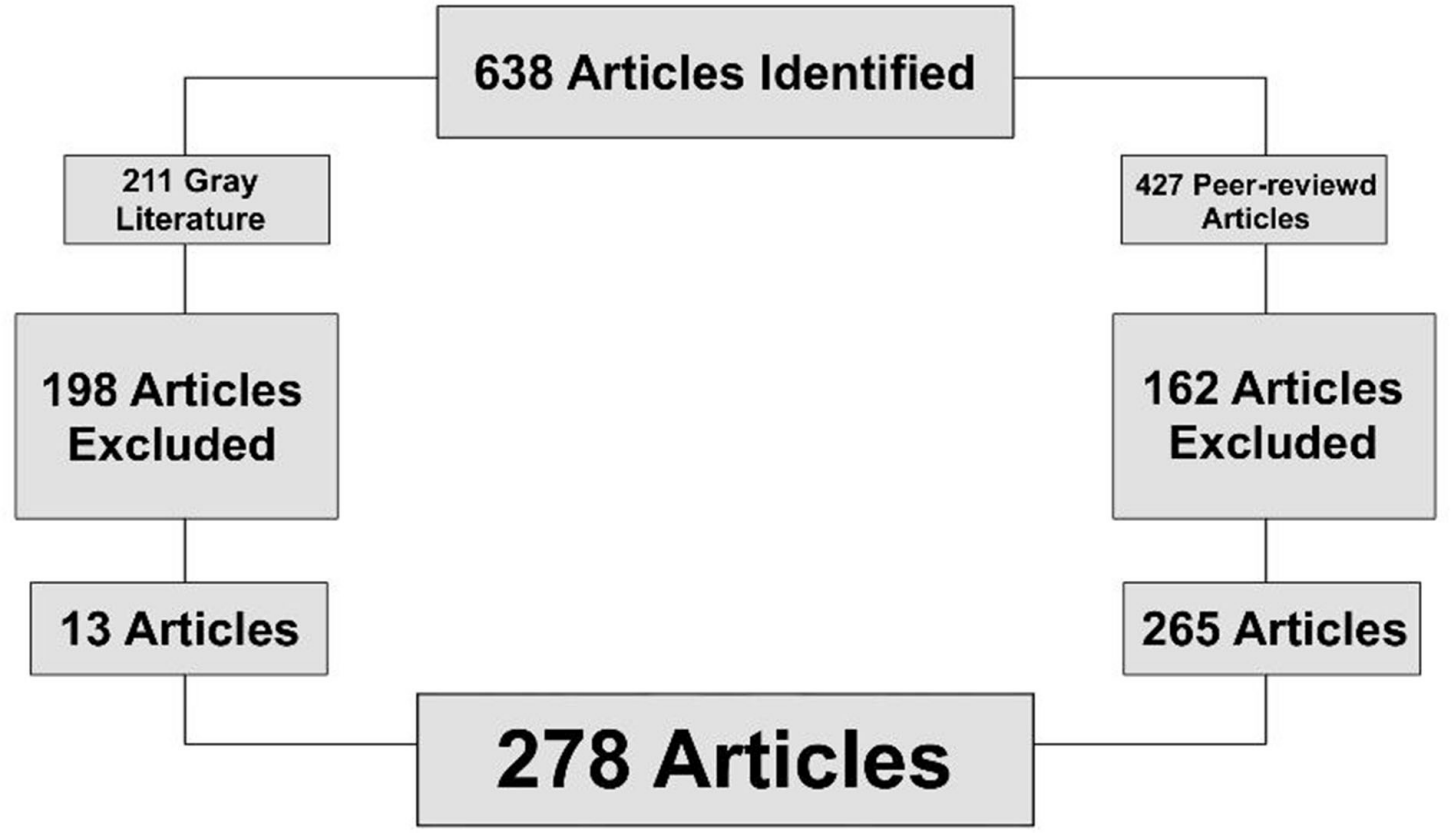
Diagram of study inclusion.

There was a total of 278 articles identified from all database searches after duplicates were removed. The articles were initially screened through application of the inclusion criteria to research titles and then to abstracts. Inclusion criteria were evaluation the mental health during pregnancy and in the time of COVID-19 pandemic. After evaluation of selected articles, a general conclusion was made based on the provided information (Figure 1).

## Mental health during the COVID-19 pandemic

The effects of the COVID-19 pandemic on mental health have various aspects. The novel COVID-19 pandemic, brings uncertainty about the future, lack of effective treatment and high mortality rate of the disease collapsed the health care systems and there was a shortage in access to mental health services.

Social support has an important role in individual's sense of belonging. Stigma toward psychiatric disorders, cause distortion in perceiving the reality and isolate the stigmatized individuals (22).

Mandatory public health policies, quarantine and mobility restrictions during the COVID-19 pandemic isolate individuals with mental disorders. Stigma and poor social support, fear of being infected and experience its complications, put pregnant women with psychiatric disorders in a vulnerable situation during the COVID-19 pandemic and they faced more social withdrawal. It affects their help-seeking behaviors and delayed in getting proper diagnosis and treatments (23).

Prolonged quarantine and social distancing, rapid and inevitable changes in the cultural and spiritual rituals cause excessive frustration, poor sleep hygiene and develop maladaptive behaviors to overcome existing stress like excessive cigarette smoking and alcohol consumption, lead to feelings of anger, discomfort, despair and a dramatic rise in domestic violence rate (24–26).

Studies showed the correlation between depressive symptoms and the COVID-19 diagnosis. People feel conflicted about following the preventive protocols and experienced complicated ambivalent state, prolonged duration of the quarantines and socioeconomic instability reduced compliance to effective preventive behaviors, which play a significant role in restricting the spread of disease, and studies have emphasized the importance of mental health in preventive behaviors (27, 28).

## Mental health in pregnancy and postpartum

The COVID-19 pandemic had a great negative impact on the utilization of maternal mental health. Even under the best of circumstances, women may be notably susceptible to mental health disorders, pregnant and postpartum women during the crisis experience more depressive and anxiety symptoms (29).

Barriers to accessing medical health care services during the COVID-19-pandemic, fear of infected from the health care systems, transportation difficulties, stigma and social labeling, lack of personal protective equipment (PPE), long waiting times at hospitals, lack of proper medication and treatment plan to treat the COVID-19 patients, and no available data about the safety of the medications and vaccine on pregnant women and their possible side effects on fetus (30).

Kingston et al., reported that stigma, lack of knowledge, and prefer to home remedy to decrease their symptoms instead of referring to clinics are major barriers that decrease the help-seeking behaviors (31).

Maternal psychiatric symptoms may adversely affect obstetrical outcome and development of the offspring. Changes in appetite and malnutrition, poor self-care and poor preventive behaviors to protect herself from trauma, self-injurious behaviors, death wish and suicidality may increase. Although, dysregulation of Hypothalamic-Pituitary-Adrenal (HPA) Axis during pregnancy contribute to the stress-related psychological and physiological responses like elevations of the cortisol level, Corticotropin-releasing hormone *(CRH)*, catecholamines have been correlated to blood flow and may lead to low birth weight, preterm delivery, long term cognitive-behavioral neurodevelopmental effects on offspring, poor weight pain during pregnancy and prenatal complications (32–34).

## Economic pandemic effects

Regarding the undeniable relation between social security, economic status, and community health, many people have become more vulnerable to the mental health problems than before, especially those with the lower socioeconomic situation and they are at a higher risk of having symptoms of depression and anxiety. The COVID-19 pandemic is not only a public health crisis but also has profound multidimensional effect on every aspect of society, especially in developing countries. Governments, businesses and individuals have been pushed to adapt rapidly. It puts the world in a “real economic freeze” state. Studies have reported that individuals who experienced economic shocks during the COVID-19 pandemic, were more likely to face mental health problems. Individuals with lower socioeconomic status and poor problem-solving styles and insufficient coping mechanisms are at a greater risk of experiencing depressive and anxious symptoms (35).

## Physical health impacts

During the pandemic, individuals experienced fear about their own and others' health; uncontrolled concerns about the consequences of disease and death of the loved one's and its unknown impacts on their children's future life that increased feeling perpetually overwhelmed and frustrated. According to a study, 83.3% of pregnant women were worried about their close family member's health, 66.7% were concerned about their older children, and 63.4% were concerned about their fetuses (36).

Forty seven percent of pregnant women reported they experienced extreme fear of their fetus structural damage following the COVID-19, and increased risk of cesarean section, low birth weight and preterm birth in pregnant women with COVID-19. A study compared pregnant women admitted to the intensive care unit (ICU) with healthy pregnant women. Preterm delivery was higher in women who were diagnosed or suspected of COVID-19 disease (19).

## Social impact

Social communications play an important role in sense of wellbeing and enhancement of mental health (10, 21). Quarantine suddenly cut off social communication, daily activities, and access to resources that usually make life easier such as mental health services. This issue has led to the 24-h presence of family members at home. As well, the closure of care centers and schools, and distance education forced parents to take care of their children all the time at home (20, 37). The cancellation of family gatherings and the impossibility of going on holidays and religious ceremonies, birthday parties, and other occasions make concern and cause a feeling of loneliness which is accompanied by a loss of motivation and a sense of peace due to being away from supportive circles (37).

## Quarantine policies

Following the government's policies to control the spread of the disease, mandatory public health policies, mobility restrictions, and stay-at-home orders to reduce the transmission had been implemented in many countries. In-person meetings were diminished and the virtual meetings and use of various social media became common. Internet Rumors and misinformation during the COVID-19 Pandemic cause confusion and misunderstanding and was positively associated with anxiety (23). Nanjundaswamy et al., reported that 40.68% of pregnant women complained about social media messages during the COVID-19 pandemic (38).

### Recommendations to improve mental health in pregnancy

#### Providing accurate information

In time of crisis, lack of accurate information from the official authorities raised prevalence of stress-related emotions during the COVID-19 pandemic and brings uncontrollable confusion, mistrust and anxiety (39). In a study comparing pregnant women undergoing treatment for COVID-19 and women without the disease, depression and anxiety scores in both groups showed significant increase during the peak of the spreading, which significantly decreased after the publication of official and accurate information about this disease through reliable sources. Governments must control the spread of pandemic news and prevent the diffusion of misinformation and gossips. By building a bridge between research and academia toward society, policy makers could achieve to solutions to the COVID-19 Misinformation Prophylaxis (23, 26).

#### Physical activity by observing health protocols

The role of physical activity on mental health is undeniable especially during pregnancy. Regular exercise reduces anxiety symptoms in pregnant women. Women who had at least 150 min of moderate exercise per week experienced less anxiety and depression compared to others (40, 41). Therefore, arrangements should be made so that pregnant women can exercise indoors or outdoors. Exercise at home can be done using TV shows, training videos, and sports apps and the importance of it should be advocate by healthcare providers (42, 43).

#### Keep in touch with friends and relatives

At the beginning of the epidemic, the rules of “social distancing” were announced, which was later replaced by the word “physical distancing”, because the importance of maintaining social relationships despite being far away is crucial in strengthening the quality of life and mental health. Interruption of interpersonal relationships lead to physical, emotional, and mental dysfunction (32). Making regular phone or video calls to family, friends, or co-workers can play an important role in reducing anxiety and loneliness and enhance sense of belonging. Partner Support During Pregnancy must be considered. Supportive partner relationship may contribute to have a great impact on maternal and infant wellbeing (44).

#### Social support systems and crisis lines

Proper and comprehensive social support reduces the long-term harmful effects of the COVID-19 pandemic during pregnancy. Constructing user-friendly crisis hotlines and publicizing reliable and scientific facts can play a role in reducing harmful thoughts and maladaptive behaviors (45).

#### Providing medical services and follow-up for pregnant women with chronic diseases

There is evidence that mental health problems are more common among pregnant women who have chronic diseases. Regarding the lack of proper access to follow-up services in patients with chronic disorders and the consequences of not receiving necessary care, it is important to provide telephone or online health services to answer individual's questions and provide them with the medical services and psycho educate them to reduce their worries and anxieties (46).

#### Early diagnosis and treatment

It is important to diagnose psychiatric disorders as soon as possible. Symptoms of psychiatric problems and red flags should be declared to pregnant women during routine prenatal visits and an effective screening system must be created to detect at risk individuals. Prenatal care should contain mental health services and provide these for all pregnant women by telephone or online contact or home visit.

#### Destigmatizing

stigmatized individuals might have constant concerns about what others think about them and preoccupied with others' opinion, and this labeling cause distortion that affect stigmatized individual's life in a wide range of activities and everyday interpersonal, occupational and social interactions and manipulate their help-seeking behaviors that cause an avoidance to refer to medical units and use proper medical and social facilities (47).

#### Providing tele-mental health services

To reduce the risk of transmitting the disease, mental health services are reduced and limited to emergencies. The need to pay attention to newer means of communication and providing telepsychiatry is felt more than ever. In various studies, telepsychiatry has had similar efficacy to face-to-face treatment. It is possible to use teleconsultation services using phone calls, web-based calls, or e-mails (48, 49).

#### Providing group therapy

Group therapy on sociological and psychological issues is one of the effective ways to reduce the distresses. Sense of being a member of a group, enhance individual's sense of We-ness and it would protect them from social isolation and can strengthen positive habits such as preventive strategies and increase hope and motivation in individuals. Group training provides the ability to transfer an enormous amount of information in a short time (50). Attending in the group activities help people to learn and develop social skills from peers, to increase their confidence and competence, promotes socialization and communication skills, reduces anxiety and loneliness (51).

#### Training classes dedicated to the challenges of pregnancy and childbirth

Due to hospital limitations and the need to reduce unnecessary procedures, 21.4% of pregnant women changed their delivery method. Changes in strategies during the pandemic have influenced decisions made during pregnancy. The most important changes have centered on hospital selection, the timing of antenatal care and delivery time, and breastfeeding patterns. These changes indicate the need for related and specialized advice. So, online consults may be a productive replacement to reduce women's stress (52).

#### Strengthening spiritual behavior

COVID-19 pandemic cause dramatic changes in individual's life and manipulate their belief systems, it is vital to consider spirituality as one of the main components of wellbeing. During the COVID-19 era, people face numerous challenges about illness, grief, regret, shame and mourning have become part of people's lives. Maintaining cultural rituals and spirituality empower people to deal with suffer by giving you a sense of peace, purpose, and forgiveness. Performing thanksgiving and religious rituals can increase resilience and perseverance. These practices increase self-confidence and can improve self-efficacy. Spiritual health can train people to find meaning in daily life and to create a purposeful life, transcendence and provide a sense of security and social structure (53, 54).

#### Self-care and healthy lifestyle

Regular physical activities, a balanced diet, and healthy sleep habits can improve people's immune systems and increase satisfaction. It is crucial to pay attention to physical and mental health during a pandemic and encourage people to find a creative way to obtain a healthy lifestyle even during the crisis. Poor diet habit and malnutrition is correlate with higher inflammation and oxidative stress which leads to depression in pregnant women during the COVID-19 pandemic (55).

### Vaccination

#### Do COVID-19 vaccines safe during pregnancy?

It is one of the frequently asked questions during the pandemic. At the beginning, the accurate data about the pathogenesis of the virus was unclear and approved vaccine haven't been established, there were a global confusion about the indication of vaccination and its complication and efficiency. The COVID-19 vaccine distribution inequality and barriers to get proper vaccine for low-income countries, anti-vaccination campaigns raised in some countries, made the confusion worse. After a while American College of Obstetrical and Gynecology (ACOG) declares that pregnant women may be vaccinated for COVID-19. The National College of French Gynecologists and Obstetricians (CNGOF) suggested mRNA vaccines for pregnant women but they shouldn't be in the first trimester, finally it's strongly recommended that the COVID-19 vaccines are safe during pregnancy and vaccination during pregnancy builds antibodies that can help protect the fetus (56–58).

## Conclusion

The COVID-19 pandemic caused considerable physical and mental challenges. This issue causes more psychological problems in vulnerable groups. Addressing the mental health of pregnant women is vital because it promotes the wellbeing of the offspring and mother and creates a healthier society. Pregnant women experienced higher levels of anxiety and depression during the pandemic.

The prenatal mental distresses, and psychiatric disorders may cause poor compliance, reduce help-seeking behaviors, and neglect to take the follow up screening visits and risk of harm for mother and others. Addressing the mental health in pregnant women is crucial to prevent the consequences.

The purpose of this narrative review was to investigate the available literature on the impact of the COVID-19 pandemic on mental health in pregnant women and provide some recommendations to improve mental health in them. It also shed some light on providing mental health services for women during pregnancy and can be used by health professionals and policymakers.

## Author contributions

All authors listed have made a substantial, direct, and intellectual contribution to the work and approved it for publication.

## Conflict of interest

The authors declare that the research was conducted in the absence of any commercial or financial relationships that could be construed as a potential conflict of interest.

## Publisher's note

All claims expressed in this article are solely those of the authors and do not necessarily represent those of their affiliated organizations, or those of the publisher, the editors and the reviewers. Any product that may be evaluated in this article, or claim that may be made by its manufacturer, is not guaranteed or endorsed by the publisher.
